# Effect of tumor genetics, pathology, and location on fMRI of language reorganization in brain tumor patients

**DOI:** 10.1007/s00330-023-09610-3

**Published:** 2023-04-19

**Authors:** Luca Pasquini, Onur Yildirim, Patrick Silveira, Christel Tamer, Antonio Napolitano, Martina Lucignani, Mehrnaz Jenabi, Kyung K. Peck, Andrei Holodny

**Affiliations:** 1 Department of Radiology, Neuroradiology Service, Memorial Sloan Kettering Cancer Center, New York, NY 10065 USA; 2 NESMOS Department, Neuroradiology Unit, Sant’Andrea Hospital, La Sapienza University, 00189 Rome, Italy; 3 Molecular Imaging and Therapy Service, Department of Radiology, Memorial Sloan Kettering Cancer Center, New York, NY 10065 USA; 4 Diagnostic Radiology Department, American University of Beirut Medical Center, Beirut, 1107 2020 Lebanon; 5 Medical Physics Department, Bambino Gesù Children’s Hospital, 00165 Rome, Italy; 6 Department of Medical Physics, Memorial Sloan Kettering Cancer Center, New York, NY 10065 USA; 7 Brain Tumor Center, Memorial Sloan Kettering Cancer Center, New York, NY 10065 USA; 8 Department of Radiology, Weill Medical College of Cornell University, New York, NY 10065 USA; 9 Department of Neuroscience, Weill Cornell Graduate School of the Medical Sciences, New York, NY 10065 USA

**Keywords:** Language, fMRI, Neural plasticity, Brain tumors

## Abstract

**Objectives:**

Language reorganization may follow tumor invasion of the dominant hemisphere. Tumor location, grade, and genetics influence the communication between eloquent areas and tumor growth dynamics, which are drivers of language plasticity. We evaluated tumor-induced language reorganization studying the relationship of fMRI language laterality to tumor-related variables (grade, genetics, location), and patient-related variables (age, sex, handedness).

**Methods:**

The study was retrospective cross-sectional. We included patients with left-hemispheric tumors (study group) and right-hemispheric tumors (controls). We calculated five fMRI laterality indexes (LI): hemispheric, temporal lobe, frontal lobe, Broca’s area (BA), Wernicke’s area (WA). We defined LI ≥ 0.2 as left-lateralized (LL) and LI < 0.2 as atypical lateralized (AL). Chi-square test (*p* < 0.05) was employed to identify the relationship between LI and tumor/patient variables in the study group. For those variables having significant results, confounding factors were evaluated in a multinomial logistic regression model.

**Results:**

We included 405 patients (235 M, mean age: 51 years old) and 49 controls (36 M, mean age: 51 years old). Contralateral language reorganization was more common in patients than controls. The statistical analysis demonstrated significant association between BA LI and patient sex (*p* = 0.005); frontal LI, BA LI, and tumor location in BA (*p* < 0.001); hemispheric LI and fibroblast growth factor receptor (FGFR) mutation (*p* = 0.019); WA LI and O6-methylguanine-DNA methyltransferase promoter (MGMT) methylation in high-grade gliomas (*p* = 0.016).

**Conclusions:**

Tumor genetics, pathology, and location influence language laterality, possibly due to cortical plasticity. Increased fMRI activation in the right hemisphere was seen in patients with tumors in the frontal lobe, BA and WA, FGFR mutation, and MGMT promoter methylation.

**Key Points:**

• *Patients harboring left-hemispheric tumors present with contralateral translocation of language function. Influential variables for this phenomenon included frontal tumor location, BA location, WA location, sex, MGMT promoter methylation, and FGFR mutation*.

• *Tumor location, grade, and genetics may influence language plasticity, thereby affecting both communication between eloquent areas and tumor growth dynamics*.

• *In this retrospective cross-sectional study, we evaluated language reorganization in 405 brain tumor patients by studying the relationship of fMRI language laterality to tumor-related variables (grade, genetics, location), and patient-related variables (age, sex, handedness)*.

**Supplementary Information:**

The online version contains supplementary material available at 10.1007/s00330-023-09610-3.

## Introduction

In the healthy population, 91–96% of right-handed subjects and 73–75% of left-handed subjects are left-hemispheric dominant for language function [1, 2]. Language reorganization has been described as a possible compensatory mechanism in response to tumor invasion of the dominant hemisphere [3]. Prior studies based on resting state fMRI (rs-fMRI) have demonstrated that both low-grade gliomas (LGG) and high-grade gliomas (HGG) can modify the functional connectivity of the language network, producing long-range effects on the right hemisphere [4]. The reorganization of language in the setting of brain tumors has been described to follow a progressive pattern, starting as intra-tumoral activation, extending to perilesional areas [5, 6], and finally leading to the recruitment of contralateral homologs [7–12]. In the case of tumors that invade eloquent language areas, functional reorganization may allow for a more complete neurosurgical resection of the tumor. Multistep surgeries have been proposed to exploit plastic changes over time to optimize tumor resection [13]. Therapies that aim to enhance language reorganization may reduce postsurgical deficits and enhance recovery [14]. However, the specific drivers of language plasticity are still unclear. Tumor type, grade, and molecular features may influence plasticity [5, 15], as they are crucial for tumor cytoarchitecture, local aggressivity, and growth. Also, damage to critical language centers that are important for subnetwork communication may cause greater effects than does damage to peripheral areas [16].

The most widely used method of preoperative evaluation of language function is task-based fMRI, which has demonstrated high specificity for language localization, and clear clinical benefits [17]. Task-based fMRI is an ideal candidate to evaluate language plasticity in brain tumors, due to its wide diffusion in the preoperative setting. This technique can provide unique insights on the functional reorganization of the language system, highlighting eloquent areas related to the language task performed in real time by the patient [18, 19]. Task-based fMRI is considered the best fMRI technique to depict language dominance and lateralization in the preoperative setting, showing high concordance with the Wada test [20], and less homotopic connectivity compared to resting-state techniques [21]. However, previous task-based fMRI studies of tumor-induced language reorganization were based on small populations or single case reports [7–10], providing limited understanding of causative mechanisms.

In this study, we used task-based fMRI to evaluate language reorganization in a large cohort of brain tumor patients. First, we investigated the effect that patient age, handedness, and sex, as well as tumor location and molecular features, have on fMRI language laterality. We studied the relationship between each of these variables and five laterality indexes (LI) calculated in the frontal lobe, Broca’s area (BA), temporal lobe, Wernicke’s area (WA), and whole cerebral hemisphere. We calculated the LI in these areas to capture language reorganization at different levels: the single-area level, evaluating the speech production hub located in BA [22] and the speech comprehension hub located in WA [22]; the lobar level, to include other relevant frontal and temporal language areas; and the hemispheric level, to include all the activation related to language tasks. The choice of calculating multiple LI was also motivated by some discrepancy regarding the best activation to depict language dominance [23, 24]. We recruited a control group of patients with tumors in the right hemisphere with the hypothesis of language reorganization being similar to the healthy population in these subjects (left dominance). We hypothesized that patients with left-hemispheric brain tumors would display more-than-expected right-hemispheric participation in language function compared to what has been previously reported in the healthy population [1, 2], while right hemispheric tumors would cause less lateralization changes. We also hypothesized that language reorganization would be associated with tumor involvement of eloquent language areas.

## Materials and methods

### Patients

This retrospective cross-sectional study was approved by the Institutional Review Board and conducted in agreement with the Declaration of Helsinki. Informed consent was waived due to retrospective design. We reviewed the imaging archive of our Institution from January 2012 to February 2022, and selected patients with the following inclusion criteria (study group): left-hemispheric brain tumors undergoing task-based fMRI for preoperative planning; no tumor involvement of the right hemisphere by either multifocal tumor or direct extension; absence of tumor-related or patient-related artifacts, including drop-out from hemorrhagic components, prior surgery, or head motion; absence of prior brain insult (i.e., stroke) or brain disease other than tumor; absence of midline shift or other major anatomic distortion from the tumor. Patients with right-hemispheric tumors were retrospectively recruited from January 2020 to September 2022 to serve as controls. The inclusion criteria for the control group were right-hemispheric brain tumors undergoing task-based fMRI for preoperative planning; no tumor involvement of the left hemisphere by either multifocal tumor or direct extension; absence of tumor-related or patient-related artifacts, including drop-out from hemorrhagic components, prior surgery, or head motion; absence of prior brain insult (i.e. stroke) or brain disease other than tumor; and absence of midline shift or other major anatomic distortion from the tumor.

Patients’ age and sex were obtained from clinical archives. Handedness was established through the Edinburgh Handedness Inventory [25]. We reviewed our pathology archive for tumor diagnosis, grade (according to the World Health Organization classification 2021 or prior), and molecular data, including isocitrate dehydrogenase (IDH) mutation, O(6)-methylguanine-DNA methyltransferase (MGMT) promoter methylation, epidermal growth factor receptor (EGFR) amplification, and fibroblast growth factor receptor (FGFR) mutation. Molecular data was obtained through the MSK-IMPACT™ (integrated mutation profiling of actionable cancer targets) test, as explained elsewhere [26]. Prior treatments including brain radiation and/or chemotherapy were retrospectively evaluated from clinical archives.

Tumor location was evaluated on MRI by three fellowship-trained neuroradiologists, with experience in fMRI. Location labels included anatomical areas (frontal, temporal, parietal, and occipital lobes; insula; and cerebellum) and language-related eloquent areas (BA, WA, Exner’s area, pre-supplementary motor area, supramarginal gyrus, and angular gyrus). Tumor size was calculated according to the response assessment in neuro-oncology (RANO) criteria, as the product of the largest perpendicular diameters of the enhancing tumor on post-contrast 3dT1-weighted images for HGG [27], or FLAIR images in the case of diffuse LGG [28]. For HGG, tumor edema was also estimated as the product of the largest perpendicular diameters of the FLAIR hyperintense component, excluding enhancing tumor and necrosis.

## fMRI acquisition

MRI acquisitions were performed on 3 T scanners (Discovery 750W, GE Healthcare) with 24-channel head coil. Functional images were acquired with a gradient-echo echo-planar imaging sequence (TR = 2500 ms; TE = 30 ms; 64 × 64 matrix; 240 mm field-of-view; 4.5 mm thickness; 80° flip angle). The standard imaging protocol included T1-weighted (TR = 600 ms; TE = 8 ms; 4.5 mm thickness) and T2-weighted (TR = 4000 ms; TE = 102 ms; 4.5 mm thickness) spin-echo axial images, and 3DT1-weighted images (TR = 22 ms; TE = 4 ms; 256 × 256 matrix; 1.5 mm thickness). Patients performed the fMRI exam with a visually administered phonemic fluency language task in which they silently generated words that began with a specific letter. This task was chosen because it is considered standard-of-care and it has proven in our experience to be robust and reliable for stimulation of language centers [29, 30]. During the task, 160 volumes of 33 images of the brain were acquired for every patient. The block paradigm used for the task included 8 cycles composed by an activation phase lasting for 20 s and a resting phase lasting for 30 s. During the activation phase, the subjects were presented with letters on a neutral background. During the resting phase, the subjects were presented with a crosshair image. Real-time software (Brainwave, Medical Numerics) was used to monitor patient task performance, associated brain activity, and head motion.

## fMRI analysis

Functional data was processed using Analysis of Functional NeuroImages [31]. Head motion was corrected using 3D rigid-body registration based on a reference volume acquired at the start. Spatial smoothing was applied using a Gaussian kernel with full width at half maximum of 4 mm to improve the signal-to-noise ratio. Removal of linear trend and high-frequency noise was performed. Cross-correlation was applied to the block paradigms in order to generate statistical parametric maps. We identified stimulus-locked responses by cross-correlating a modeled waveform corresponding to the task performance block with all pixel time courses on a pixel-by-pixel basis. To minimize false positives, voxels with standard deviation exceeding 8% of the mean signal intensity were set to zero.

To minimize inter-subject variability, we calculated multiple LIs on fMRI maps through a threshold-independent method [32, 33]. The workflow was as follows: (1) 3D T1-weighted and functional images were registered to MNI152 standard space through nonlinear registration (ANTS). The registration of functional images was based on the first volume of the echo-planar sequence and applied to the correlation maps generated after post-processing. fMRI activation maps were overlayed on structural images before and after co-registration for quality assessment; (2) co-registered 3D T1-weighted images were parcellated according to the automated anatomical labeling—AAL atlas; (3) for every subject, we calculated the mean value of the 5% most-activated voxels on the correlation map; (4) we considered the number of voxels above 80% of this mean within each region of interest (ROI) and applied the traditional formula, LI = (L-R)/(L + R), where L and R represent the number of voxels in the left and right ROI, respectively.

We calculated five LIs corresponding to the following ROIs: hemispheric ROI, excluding cerebellum and visual cortex; temporal lobe; frontal lobe; BA, including the pars opercularis and triangularis of the inferior frontal gyrus; and WA, including the posterior aspect of the superior temporal gyrus. For each subject, an experienced neuroradiologist confirmed the findings by visually comparing the LIs generated by our method to the respective fMRI maps overlayed on 3dT1 anatomical images.

## Statistical analysis

Statistical analyses were performed on SPSS (IBM Corp. Version 25.0). We set the significance threshold (*p*) for all analyses to 0.05. We created five binary variables corresponding to hemispheric LI, Broca’s LI, Wernicke’s LI, frontal LI, and temporal LI, with the cutoff of 0.2 [23]. Values of LI ≥ 0.2 were defined as left-lateralized (LL), while values < 0.2 were defined as atypical lateralized (AL), including co-lateralized and right-lateralized. Chi-square test was employed to identify the relationship between LI variables and other nominal variables, including patient sex and handedness, tumor grade, location, and genetic and molecular data. Tumor grade was defined as a variable with three values (1 = grade I/II; 2 = grade III/IV; 3 = metastases). The other variables analyzed with chi-square test were binarized (0/1 = absent/present). For those variables having significant chi-square test results, contingency coefficient, phi factor, and Cramer’s V were computed. The analysis was repeated for significant variables in subgroups of patients divided by tumor grade. The full data about tumor location and molecular markers versus laterality is provided as supplementary material in excel format.

A multinomial logistic regression analysis was performed to determine the effect of confounding factors on those variables having significant chi-square test results. In particular, we predicted the LI values using tumor location, grade, or characteristics (e.g., MGMT, EGFR, FGFR) as predictors, while age, tumor size, tumor edema, and previous chemo/radio-therapy were used as covariates. The analysis was performed in R environment with R-notebook of google Colab (colab.to/r), using *multinom* function to assess logistic regression.

## Results

**Fig. 1:**
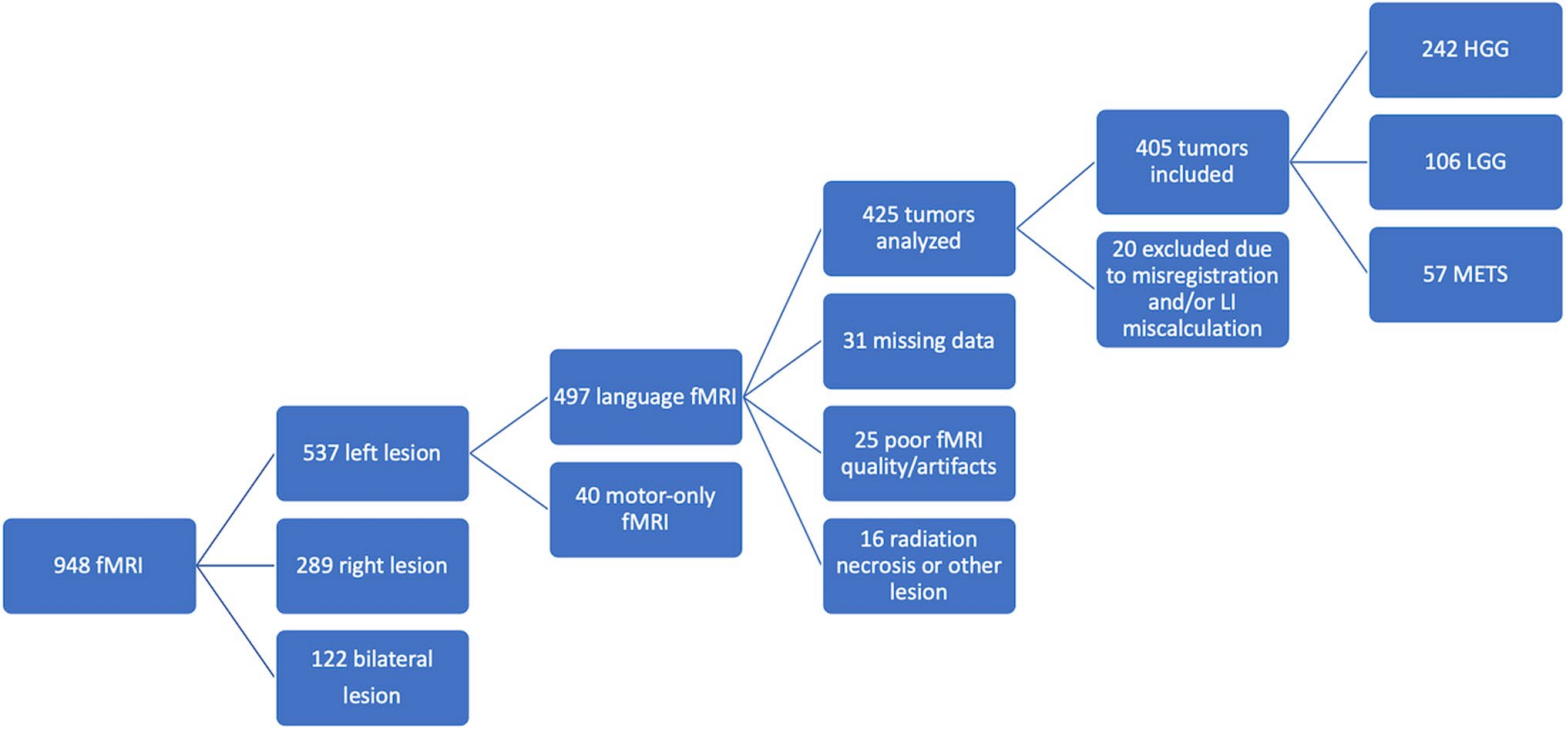
Flowchart of our study’s patient selection. HGG, high-grade glioma; LGG, low-grade glioma; LI, laterality index; METS, metastases

Four hundred and five patients were included in the study group. The recruitment steps are displayed in Fig. 1. One hundred and six patients were diagnosed with low-grade glioma (LGG, WHO grade 1–2); 242 patients were diagnosed with high-grade glioma (HGG, WHO grade 3–4); and 57 patients were diagnosed with metastatic disease. Patient variables, tumor location, and involvement of eloquent areas are summarized in Table 1. Due to retrospective design, molecular features were only available for part of the subjects. IDH status was present in 240/405 patients (124 wild-type); MGMT status in 255/405 (144 unmethylated); EGFR in 188/405 (46 amplified); and FGFR in 198/405 (16 mutated). Characteristics of tumor genetic sub-groups are summarized in Table 2.

**Table 1:** Demographic data and tumor location

	HGG	LGG	METS
Age (mean and SD)	53 ± 14	43 ± 13	54 ± 13
Sex	152 M; 90 F	56 M; 50 F	27 M; 30 F
Handedness	216 R; 26 L	92 R; 14 L	53 R; 4 L
Frontal lobe	126	59	37
Temporal lobe	88	40	12
Parietal lobe	65	14	18
Occipital lobe	8	2	2
Insula	45	36	1
BA	54	21	2
EA	28	19	3
SMA	30	12	8
WA	30	5	5
SMG	16	3	6
AG	18	3	4

*AG* angular gyrus, *BA* Broca’s area, *EA* Exner’s area, *SMA* pre-supplementary motor area, *SMG* supramarginal gyrus; *WA* Wernicke’s area

**Table 2:** Characteristics of genetic sub-groups

	IDH **+ **	IDH −	MGMT +	MGMT −	EGFR +	EGFR −	FGFR +	FGFR −
AGE (mean and SD)	45 ± 13	57 ± 14	53 ± 13	51 ± 16	60 ± 12	52 ± 13	51 ± 18	52 ± 15
SEX	70 M/46 F	76 M/48 F	74 M/37 F	82 M/62 F	34 M/12 F	82 M/60 F	8 M/8 F	112 M/70 F
HANDEDNESS	96 R/20 L	113 R/11 L	111 R/19 L	133 R/11 L	39 R/7 L	133 R/9 L	155 R/25 L	14 R/2 L
TUMOR SIZE	9.05 cm^2^	11.15 cm^2^	11.1 cm^2^	13.7 cm^2^	16.75 cm^2^	14.75 cm^2^	5.7 cm^2^	9.7 cm^2^

*EGFR* epidermal growth factor receptor amplification, *FGFR* fibroblast growth factor receptor mutation, *IDH* isocitrate dehydrogenase mutation, *MGMT* O(6)-methylguanine-DNA methyltransferase promoter methylation

Forty-nine patients with right-hemispheric tumors were recruited to serve as controls (mean age 51 ± 14 years, 36 males and 13 females, 44 right-handed and 5 left-handed). Among these subject, 26 had HGG, 16 LGG and 7 brain metastases.

**Fig. 2:**
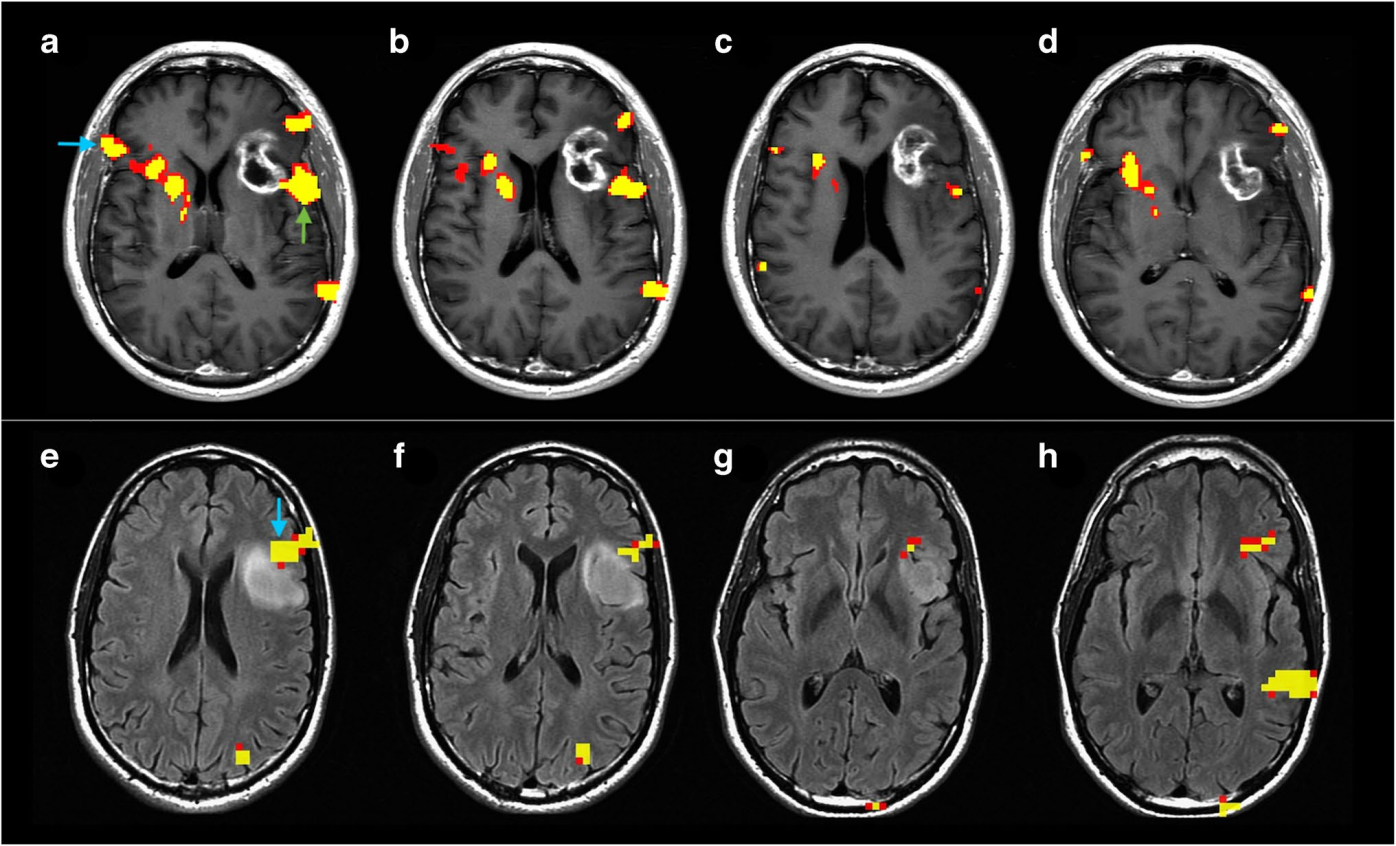
Exemplificative cases of language plasticity. Above, fMRI correlation map overlayed on axial post-contrast T1-weighted images of a 67-year-old man with glioblastoma affecting the left, lower-frontal lobe. The fMRI map shows co-dominant activation of Broca’s area (left activation: green arrow in **a**; right activation: light blue arrow in **a**). The hemispheric LI of this patient was − 0.32. Below, fMRI correlation map overlayed on axial post-contrast T1-weighted images of a 51-year-old man with glioblastoma affecting the left, lower-frontal lobe. This fMRI map shows left-dominant activation of Broca’s area (light blue arrow in **e**). The hemispheric LI of this patient was 1

**Fig. 3:**
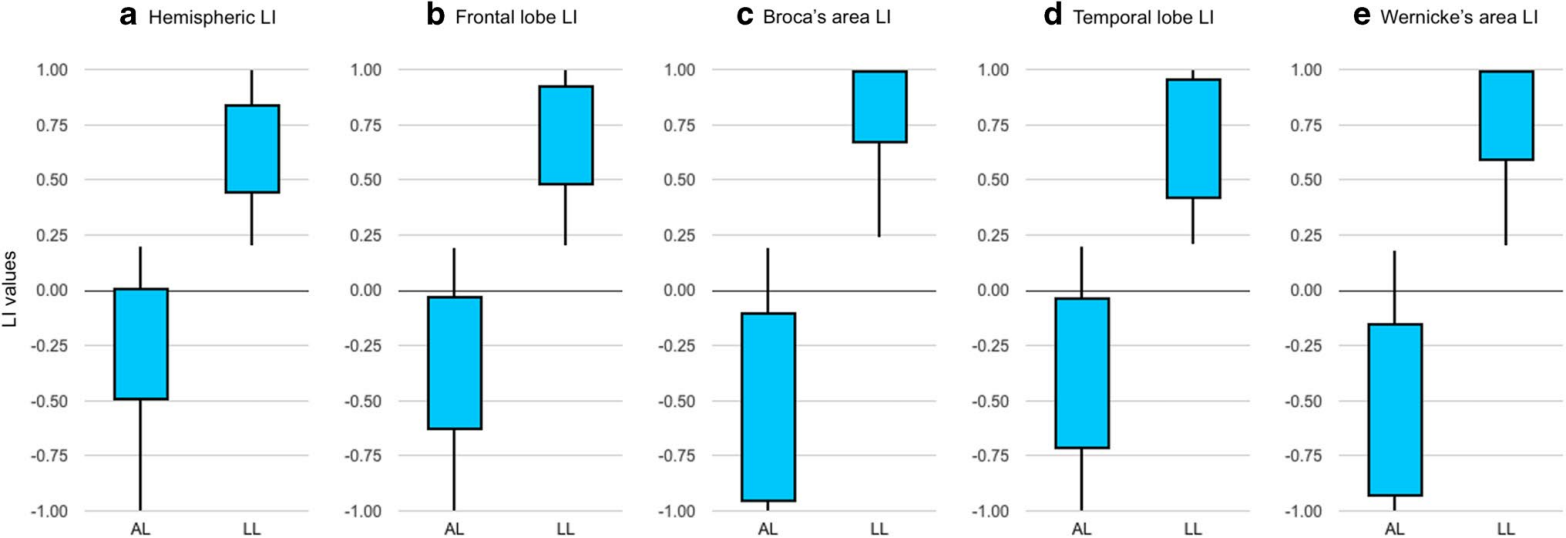
Box plots representing the distribution of the five laterality indexes (LI) calculated in the total population of our study: hemispheric LI (**a**), frontal LI (**b**), Broca’s LI (**c**), temporal LI (**d**), and Wernicke’s LI (**e**). Hemispheric LI demonstrated 210 left-lateralized (LL) and 195 atypical lateralized (AL) patients. Frontal LI demonstrated 221 LL and 184 AL patients. Broca’s LI demonstrated 234 LL and 171 AL patients. Temporal LI demonstrated 220 LL and 185 AL patients. Wernicke’s LI demonstrated 215 LL and 190 AL patients

In the study group, language lateralization based on hemispheric LI demonstrated 212 LL and 193 AL patients. Frontal LI demonstrated 217 LL and 188 AL patients. Broca’s LI demonstrated 236 LL and 169 AL patients. Temporal LI demonstrated 222 LL and 183 AL patients. Wernicke’s LI demonstrated 217 LL and 188 AL patients. Laterality results according to tumor type are reported in Table 3. Two representative cases are displayed in Fig. 2. The distribution of the five LIs is presented in Fig. 3. The age distribution of AL and LL patients according to the calculated LIs was not significantly different on Mann–Whitney *U* test.

**Table 3:** Laterality results for left-hemispheric tumor patients

	HGG	LGG	METS
Hemispheric LI			
AL	51% (123)	41% (43)	48% (27)
LL	49% (119)	59% (63)	52% (30)
Frontal LI			
AL	59% (143)	41% (43)	54% (31)
LL	41% (99)	59% (63)	46% (26)
Broca’s LI			
AL	46% (111)	35% (37)	37% (21)
LL	54% (131)	65% (69)	63% (36)
Temporal LI			
AL	57% (138)	57% (60)	42% (24)
LL	43% (104)	43% (46)	58% (33)
Wernicke’s LI			
AL	51% (123)	39% (41)	42% (24)
LL	49% (119)	61% (65)	58% (33)

*AL* atypical laterality, *HGG* high-grade glioma, *LGG* low-grade gliomas, *LL* left laterality, *METS* metastases

In the control group, language lateralization based on hemispheric LI demonstrated 35 LL and 14 AL patients. Frontal LI demonstrated 40 LL and 9 AL patients. Broca’s LI demonstrated 46 LL and 3 AL patients. Temporal LI demonstrated 31 LL and 18 AL patients. Wernicke’s LI demonstrated 41 LL and 8 AL patients. Language laterality results in the control group are summarized in Table 4 and supplementary Figs. 1–5.

**Table 4:** Laterality results for right-hemispheric tumor patients

	HGG	LGG	METS
Hemispheric LI			
AL	19% (5)	37% (6)	43% (3)
LL	81% (21)	63% (10)	57% (4)
Frontal LI			
AL	19% (5)	19% (3)	14% (1)
LL	81% (21)	81% (13)	86% (6)
Broca’s LI			
AL	8% (2)	0% (0)	14% (1)
LL	92% (24)	100% (16)	86% (6)
Temporal LI			
AL	34% (9)	37% (6)	43% (3)
LL	66% (17)	63% (10)	57% (4)
Wernicke’s LI			
AL	11% (3)	31% (5)	0% (0)
LL	89% (23)	69% (11)	100% (7)

*AL* atypical laterality, *HGG* high-grade glioma, *LGG* low-grade gliomas, *LL* left laterality, *METS* metastases

The chi-square analysis for tumor location variables demonstrated a significant correlation between frontal LI, Broca LI, and tumor involvement of BA, indicating that this location is more likely associated with AL (chi-square *p* < 0.001; Fisher *p* < 0.001). When the analysis was repeated in separate groups divided by tumor grade, this correlation remained true only for HGG (chi-square *p* < 0.001; Fisher *p* < 0.001). None of the remaining locations produced significant results. Additionally, HGG located in BA and not extending to the temporal lobe (temporal lobe spared) demonstrated a significant correlation to temporal LI (chi-square *p* = 0.032; Fisher *p* = 0.048), indicating that BA location is more likely associated with temporal AL. Similarly, HGG located in the temporal lobe and not extending to the frontal lobe demonstrated a significant correlation to Broca LI (chi-square *p* = 0.019; Fisher *p* = 0.027), suggesting that temporal location is associated with Broca AL.

Pathology data analysis demonstrated a significant correlation between tumor grade, Broca LI, and hemispheric LI (chi-square *p* < 0.001; Fisher *p* < 0.001), with higher grades showing greater association to AL. Hemispheric LI showed a significant correlation to EGFR amplification in all tumors, with amplification being more likely associated with AL (chi-square *p* = 0.042; Fisher *p* = 0.05). FGFR mutation correlated significantly with hemispheric LI in all tumors and was more likely to be associated with LL (chi-square *p* = 0.019; Fisher *p* = 0.021). Wernicke LI correlated with MGMT status, but only for HGG, and AL showed greater association to hypermethylation (chi-square *p* = 0.016; Fisher *p* = 0.014).

Analysis of remaining variables demonstrated a significant correlation between patient sex and Broca LI (chi-square *p* = 0.005; Fisher *p* = 0.001), with female sex more likely to be associated with AL. Patient handedness did not show any significant correlations.

The multinomial logistic regression model confirmed the statistical significance of the majority of our predictions. Using tumor involvement of BA as predictor, we were able to accurately predict Broca LI (*p* = 1.55e^−05^) and frontal LI (0.002) when considering all tumor grades. This evidence remained true for the HGG group when we separated groups by tumor grade (*p* = 4.27e^−05^ and *p* = 0.001 respectively for Broca and frontal LI). Moreover, significant results for Broca LI prediction were also found for the HGG group located in the temporal lobe (*p* = 0.007). When FGFR mutation was used as predictor, highly significant results were found for hemispheric LI prediction (*p* = 0) for all tumor grades. Finally, Wernicke LI prediction resulted when MGMT promoter methylation was used as predictor for the HGG group (*p* = 0.027). Conversely, tumor grade and EGFR amplification did not show any predictive performance on language laterality in the model. Prior chemotherapy treatment was a significant covariate between Broca’s LI and tumor grade. Age, tumor size, tumor edema, and prior radiotherapy did not show significant influence on the results (Supplementary Table 1 and 2).

## Discussion

Patients with left-hemispheric brain tumors performing language fMRI presented more right-hemispheric activation than expected from previous studies in the normal population [1, 2]. On the other hand, patients with right-hemispheric tumors were predominantly left-dominant. These findings support the idea of tumor-induced language reorganization in patients with left-hemispheric brain neoplasms. Holodny et al reported for the first time the translocation on fMRI of BA to the right hemisphere in a patient with left-hemispheric glioma [8]. Other authors reported similar findings for frontal and temporal language areas in small populations of brain tumor patients [7, 9, 10, 34, 35]. The presence of inter-hemispheric language reorganization has been confirmed using magnetoencephalography (MEG) [36], positron emission tomography [37], and transcranial magnetic stimulation [38]. The neurobiological mechanisms of language plasticity are still under investigation. Changes of brain function may lead to structural modifications or vice-versa because intense neurotransmitter release regulates the number of local cells or synapses [39]. This concept is supported by the evidence of learning-dependent increases of cortical volume for linguistic, procedural, spatial orientation, and navigation abilities [40–42], whose etiology may depend on a combination of increased cell size or spine density, neurogenesis, myelin plasticity, axonal sprouting, or angiogenesis. Previous studies demonstrated that language activations in the right hemisphere of patients with brain tumors are associated with increased cortical volume, confirming the presence of underlying structural changes of the cortex [43]. In this study, we demonstrated language reorganization in the largest sample of brain tumor patients to date, highlighting the capability of task-based fMRI to detect plastic changes. We demonstrated that only left-sided tumors cause inter-hemispheric reorganization of language, supporting the idea that language reorganization represents an attempt of the brain to compensate the cognitive deficit caused by a tumor in the dominant language areas. Furthermore, we demonstrated associations of language reorganization with tumor location and genetics. These findings are particularly important in light of the wide use of task-based fMRI in preoperative planning and the possibility of selecting eligible patients for tailored surgery [13, 14] or future plasticity-enhancing therapies [14].

The highest percentage of tumors with right shift in laterality was seen among HGG (Table 2). This result could be related to two main factors: (1) the presence of neuro-vascular uncoupling (NVU), which is much more common in high-grade tumors, leading to decrease of perilesional BOLD signal [44]; (2) the development of language reorganization to the right-hemisphere (inter-hemispheric reorganization). To test these hypotheses, we compared the laterality shift on the frontal and temporal LI. Our results demonstrate that language activation shift in HGG is not confined to the tumor’s location in the cerebral lobe. In fact, frontal HGG affecting BA were associated with right-shift of temporal LI. Similarly, temporal HGG were associated with Broca’s LI right-shift. Although NVU can be considered a problem of the entire brain, its effect is usually maximal in the region of the tumor and progressively decreases in nearby and distant regions [45]. Our findings may point to the presence of inter-hemispheric reorganization, which extends beyond the local effects of NVU on the BOLD signal. In this view, these results may support the contention that the observed cortical reorganization is “true” rather than “pseudo-reorganization” caused by NVU. Previous authors reported language plasticity in mixed populations of LGG and HGG [10, 35]. Traut et al used MEG to investigate language plasticity in patients affected by glioma, showing that both low- and high-grade lesions may trigger functional reorganization [36]. Our results seem to confirm this idea in HGG. Nevertheless, a definite conclusion cannot be drawn at this point.

Few reports exist regarding the effect of specific tumor locations on language reorganization [3, 11]. We found a significant correlation between frontal and Broca’s LI, and HGG location in BA. Evidence from intra-operative cortical stimulation suggests that BA is prone to plasticity and may reorganize to the nearby frontal or insular cortex [5]. Other authors reported that tumors in frontal areas are more prone to result in cortical reorganization than those in temporal areas [11]. Our findings support the plasticity of BA in the form of inter-hemispheric reorganization to the contralateral homolog.

We demonstrated that brain tumor genetics have a significant correlation with language laterality. Molecular features strongly influence the tumor cytoarchitecture, growth, pattern of spread, and interaction with surrounding areas. MGMT promoter methylation was associated with more right-hemispheric activation in HGG. This may be explained by the survival advantage of MGMT hypermethylation [46], which provides more time to develop plasticity. EGFR amplification was associated with right-shift of language activation in the chi-square analysis, although without reaching statistical significance in the logistic regression model. This genetic abnormality is related to tumor vascularity and grade [47], while increased tumor perfusion is predictive of EGFR amplification [48]. Some influence of EGFR mutations on language lateralization could be explained with the correlation between increased vascularization (typical of EGFR amplification) and NVU [44], which leads to the suppression of intra- and peritumoral BOLD signal (“pseudo-reorganization”). The lack of FGFR mutations or rearrangement was associated with right-shift in laterality. FGFR abnormalities are oncogenic by promoting tumor proliferation and migration [49]. Somatic mutations of FGFR are frequent in glioblastoma, supporting both tumor growth and progression [49]. Increased proliferation and tumor activity related to FGFR abnormalities may limit the plastic potential of the brain.

The age distribution between AL and LL patients was not significantly different in our groups, and no age-related effect emerged from our analyses. A change in functional reorganization depending on patients’ age may be expected based on previous literature. In fact, the plastic potential of the brain progressively diminishes with age [50]. Plasticity related to function-learning is less pronounced in older subjects, and neurogenesis may also be limited [50, 51]. Similarly, the ability of the brain to compensate for tumoral invasion of the dominant hemisphere through inter-hemispheric language reorganization may decrease with age. Future studies with a broader patient age range are needed to clarify this aspect.

Patients’ handedness did not show significant effects on language laterality in our analyses. This observation may suggest that, in the setting of brain tumors, other variables come into play to affect language laterality, further strengthening the idea of tumor-induced plastic changes rather than native AL in our study. The marginal role of handedness in this study may also be due to statistical reasons, since our population was predominantly right-handed (above 89%) and therefore not ideal to infer handedness-related effects.

Finally, although the prediction of Broca’s LI from tumor grade was not significant, prior chemotherapy proved to be a significant covariate in the statistical model for this association (supplementary Table 1). Particularly, prior chemotherapy was associated with right-lateralized Broca’s LI. Chemotherapy may lead to structural and functional changes in the brain, which persist over time [52]. Brain plasticity in the setting of systemic treatments may represent a compensatory mechanism to counteract the detrimental effects of chemotherapy. Future studies could further explore this hypothesis.

Our study has some limitations. Due to its retrospective design, only part of our patient cohort had available clinical information. We were therefore unable to collect enough data for meaningful comparison, particularly regarding patients’ language performance. Genetic and epigenetic information was also limited for similar reasons. Future studies are needed to confirm our results and to explore the impact of language reorganization on patients’ clinical deficits. Finally, we employed an operator-independent and threshold-independent method to assess language laterality based on previous studies [32]. This choice limits the comparability of our results with those obtained using different methods.

## Conclusion

Our results demonstrate that patients harboring left-hemispheric tumors present with significant fMRI activation in the right hemisphere, indicating the translocation of language function due to cortical plasticity. This phenomenon is well observed in HGG, LGG, and metastases. Particularly, frontal tumors and the involvement of BA seem to favor reorganization. Genetic and molecular tumor features appear to play a role in the development of laterality changes.

## Supplementary Information

Below is the link to the electronic supplementary material.

Supplementary file1 (RAR 291 KB)

## Abbreviations

AL: Atypical lateralized
BA: Broca’s area
EGFR: Epidermal growth factor receptor
FGFR: Fibroblast growth factor receptor
HGG: High-grade glioma
IDH: Isocitrate dehydrogenase
LGG: Low-grade glioma
LL: Left lateralized
MEG: Magnetoencephalography
MGMT: O6-Methylguanine-DNA methyltransferase
NVU: Neuro-vascular uncoupling
ROI: Region of interest
WA: Wernicke’s area

## Declarations

### Guarantor

The scientific guarantor of this publication is Luca Pasquini.

### Statistics and biometry

The author Antonio Napolitano has significant statistical expertise, and provided statistical advice for this manuscript.

### Methodology

The design of the study was retrospective cross-sectional performed at one institution.
